# Supplementary material for: A co-ordinated transcriptional programme in the maternal liver supplies long chain polyunsaturated fatty acids to the conceptus using phospholipids
Source: Nat Commun. 2024 Aug 8;15:6767. doi: 10.1038/s41467-024-51089-z (PMC11310303; doi:10.1038/s41467-024-51089-z)
Supplement: Supplementary file 1 — Supplementary Information [file 41467_2024_51089_MOESM1_ESM.pdf]

Supplementary Figure 1

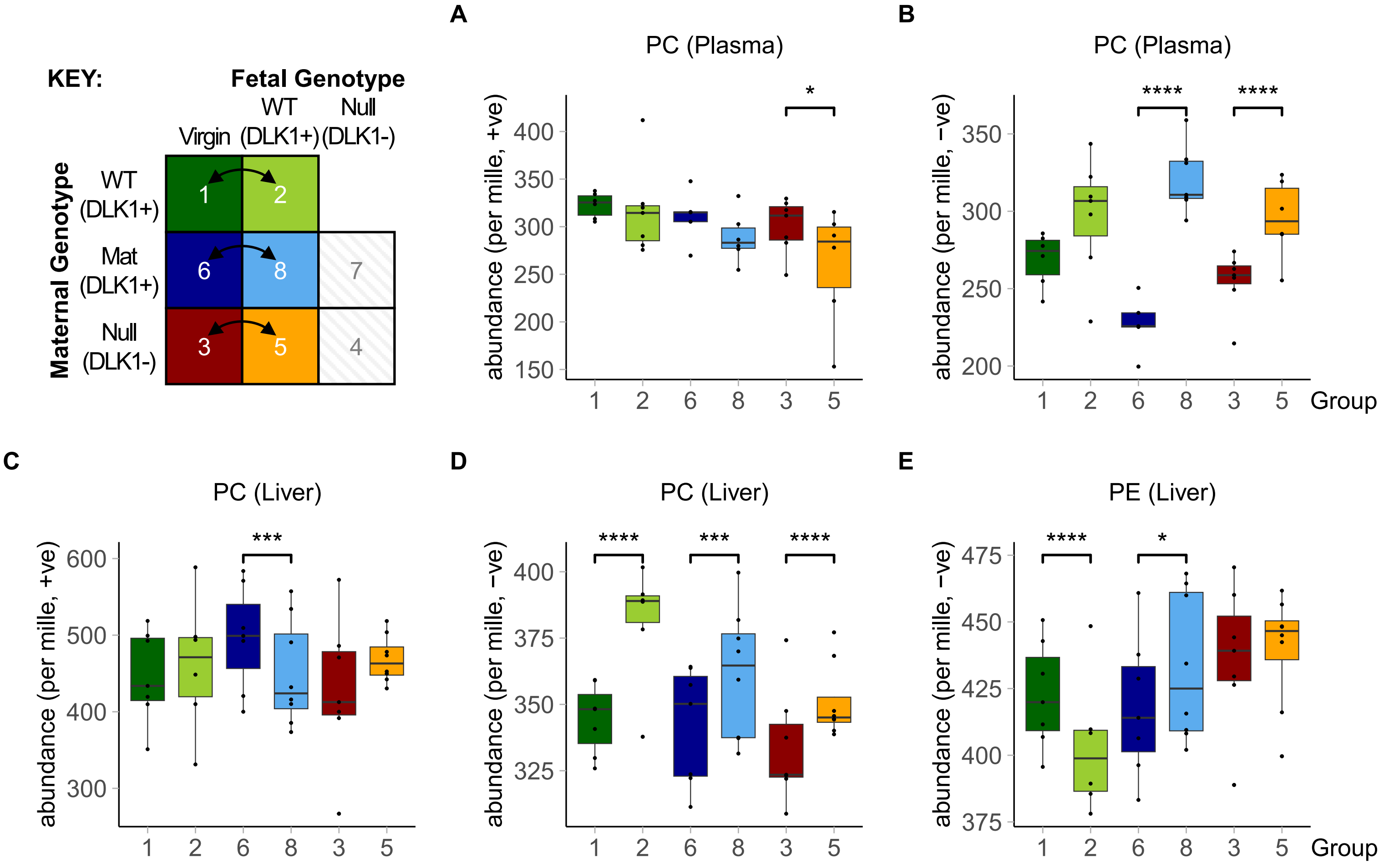

Figure S1: Grouped relative abundance of PC in plasma **(A-B)** and liver **(C-D)** and PE in liver **(E)** in three genotype-matched virgin vs pregnant (15.5 dpc) replicate group comparisons. PC data is shown from both positive and negative ionisation modes. Data is presented as boxplots (median and IQR (25th and 75th percentiles) with whiskers showing 1.5*IQR) with individual values. Two-way ANOVA with Sidak's multiple comparisons tests were performed to determine significant class shifts between experimental groups (* p-value <0.05; ** p-value <0.01; *** p-value <0.001; **** p-value <0.0001). Statistical tests were performed independently per ionisation mode and per replicate comparison. p-values for A: 1vs2 = NS, 6vs8 = NS, 3vs5 = 0.025; B: 1vs2 = NS, 6vs8 = $1.00x10^{-15}$, 3vs5 = $6.37x10^{-10}$; C: 1vs2 = NS, 6vs8 = 0.0001, 3vs5 = NS; D: 1vs2 = $1.00x10^{-15}$, 6vs8 = 0.0001, 3vs5 = $7.38x10^{-05}$; E: 1vs2 = $9.41x10^{-06}$, 6vs8 = 0.014, 3vs5 = NS. Plasma data: n = 6 (group 1), n = 7 (group 2), n = 7 (group 3), n = 6 (group 5), n = 5 (group 6), n = 6 (group 8; +ve mode), n = 7 (group 8; -ve mode); Liver data: n = 7 (group 1), n = 6 (group 2), n = 7 (group 3), n = 8 (group 5), n = 7 (group 6), n = 8 (group 8); mice per group PC, phosphatidylcholine; PE, phosphatidylethanolamine. Source data are provided as a Source Data file.

Supplementary Figure 2

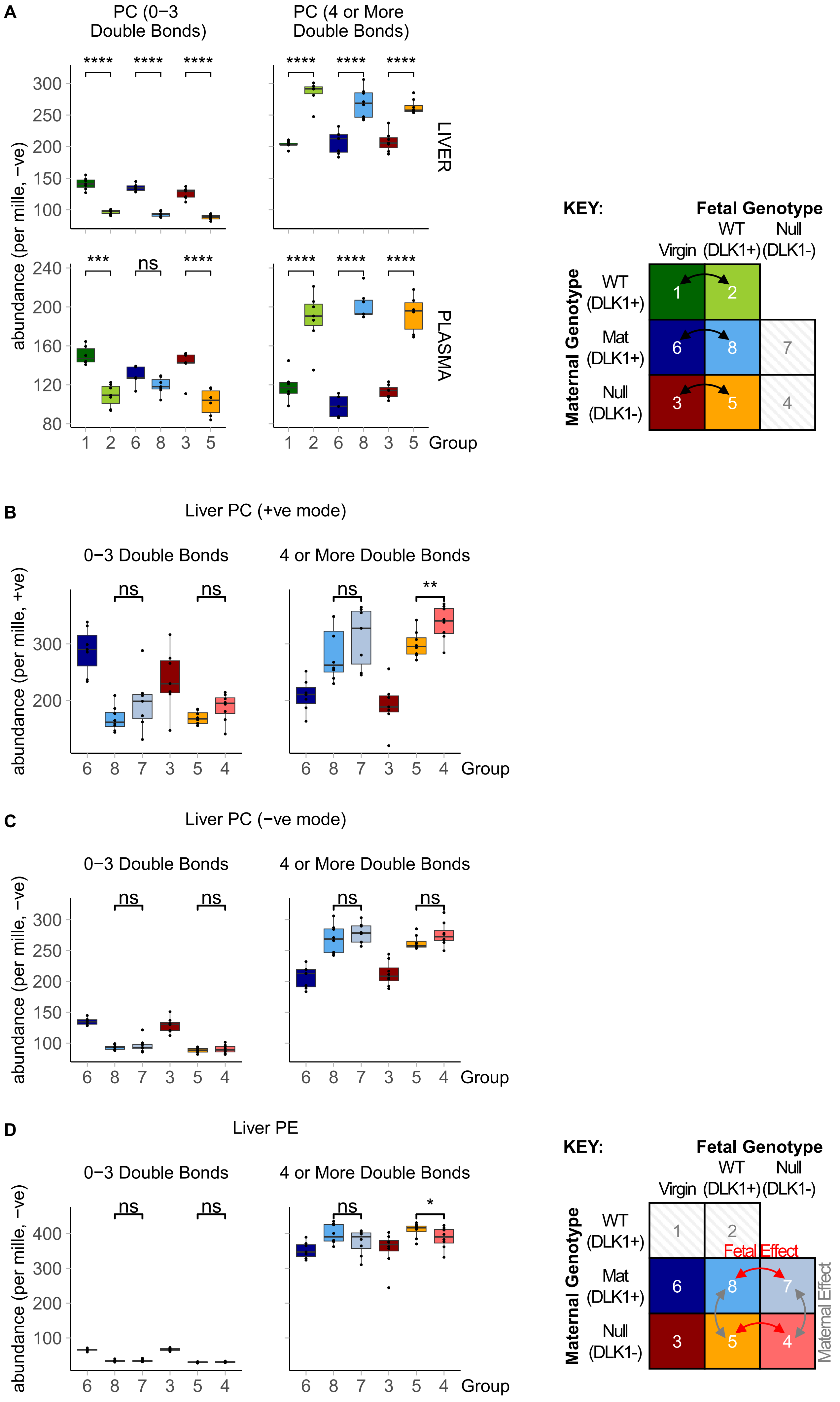

Figure S2: (A) Grouped relative abundance of PC lipids measured in the negative ionisation mode that contain fatty acids with a combined total of three or fewer double bonds (left) or four or more double bonds (right) in the liver and plasma of virgin and pregnant (15.5 dpc) groups. **(B-D)** Grouped relative abundance of PC **(B-C)** and PE **(D)** lipids that contain fatty acids with a combined total of three or fewer double bonds (left) or four or more double bonds (right) in livers of pregnant mice that lack fetal or maternal-derived DLK1 protein. Significance was only considered if identified in at least two genotype-matched replicate group comparisons. Data is presented as boxplots (median and IQR (25th and 75th percentiles) with whiskers showing 1.5*IQR) with individual values and two-way ANOVA with Sidak's multiple comparisons were performed for each genotype-matched comparison (* p-value <0.05; ** p-value <0.01; *** p-value <0.001; **** p-value <0.0001). All statistical tests were performed independently per ionisation mode and per replicate comparison. p-values for A - Liver PC (0-3 Double Bonds): 1vs2 = 5.40x10-07, 6vs8 = 3.02x10-05, 3vs5 = $8.39x10^{-07}$; A - Liver PC (4+ Double Bonds): 1vs2 = $1.12x10^{-11}$, 6vs8 = $5.81x10^{-08}$, 3vs5 = $7.81x10^{-10}$; A - Plasma PC (0-3 Double Bonds): 1vs2 = 0.0008, 6vs8 = NS, 3vs5 = $9.62x10^{-05}$; A - Plasma PC (4+ Double Bonds): 1vs2 = $1.05x10^{-06}$, 6vs8 = $3.04x10^{-12}$, 3vs5 = $1.71x10^{-09}$; B - Liver PC (4+ Double Bonds): 5vs4 = 0.006; Liver PE (4+ Double Bonds): 5vs4 = 0.05. Plasma data: n = 6 (group 1), n = 7 (group 2), n = 7 (group 3), n = 6 (group 5), n = 5 (group 6), n = 6 (group 8; +ve mode), n = 7 (group 8; -ve mode); Liver data: n = 7 (group 1), n = 6 (group 2), n = 7 (group 3), n = 8 (group 4), n = 8 (group 5), n = 7 (group 6), n = 7 (group 7, +ve mode), n = 8 (group 7, -ve mode), n = 8 (group 8); mice per group. PC, phosphatidylcholine; PE, phosphatidylethanolamine. Source data are provided as a Source Data file.

Supplementary Figure 3

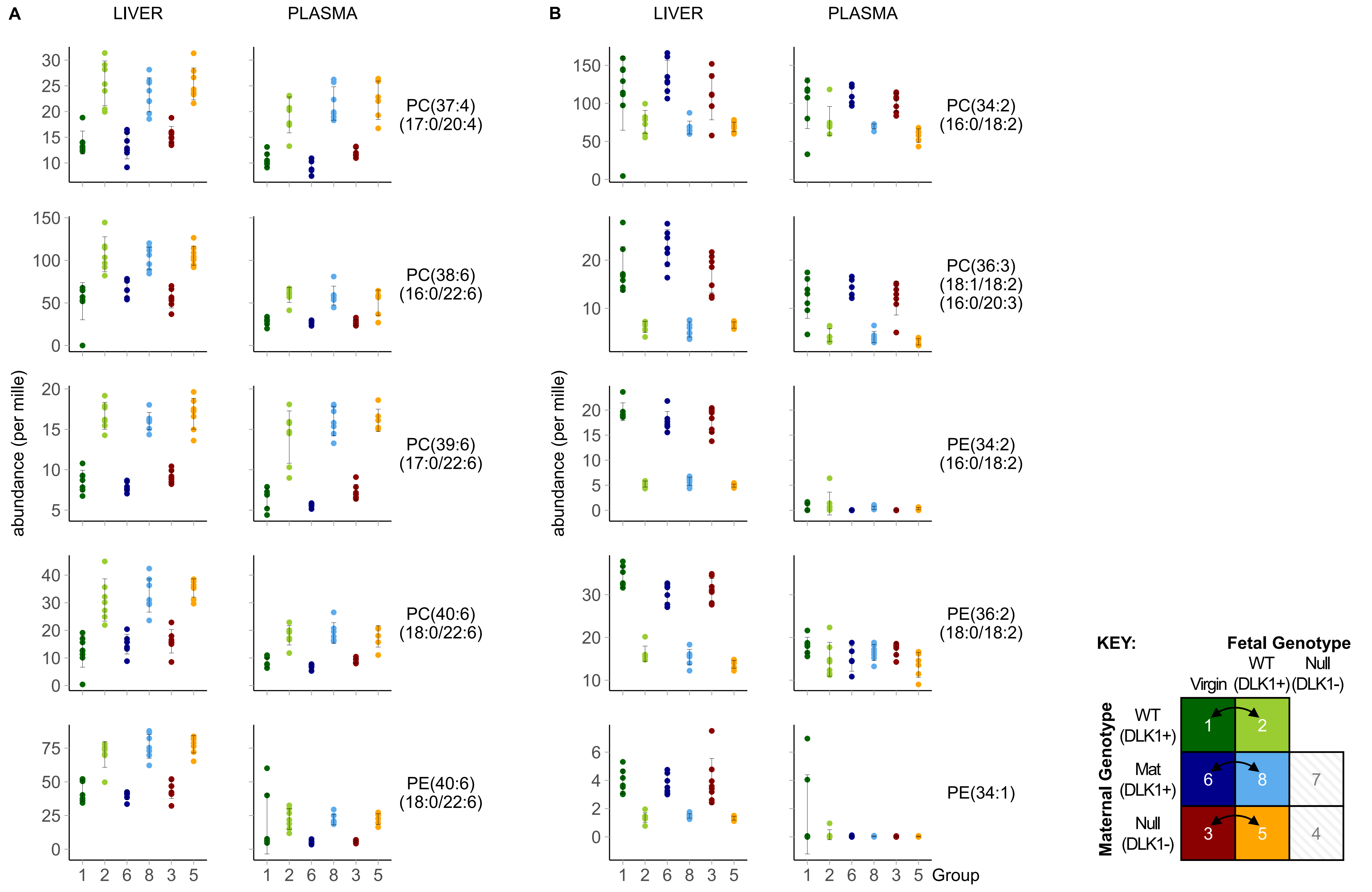

Figure S3: PC or PE lipids of interest that contain an *sn*-2 fatty acid with four or more double bonds **(A)** and three or fewer double bonds **(B)** that were identified as candidate biomarkers (CBMs) associated with pregnancy. CBMs are classified as lipids that passed both Bonferroni-adjusted two-tailed *t*-tests (liver threshold, p = 0.00234; plasma threshold, p = 0.00283) and sparse partial least squares discriminant analysis in at least two genotype-matched replicate virgin vs pregnant (15.5 dpc) comparisons (see Supplementary Table S4 for full CBM list). CBM data is shown as relative abundance per ionisation mode with ± SD error bars. *sn*-1/*sn*-2 fatty acid compositions were assigned using the most abundant isoform identified from targeted LC-MS/MS analysis in plasma and liver (Supplementary Table S6). All CBM tests were performed independently per ionisation mode and per genotype-matched replicate comparison. Plasma data: n = 7 (group 1), n = 7 (group 2), n = 7 (group 3), n = 6 (group 5), n = 5 (group 6), n = 7 (group 8); Liver data: n = 8 (group 1; +ve mode), n = 7 (group 1; -ve mode), n = 7 (group 2), n = 7 (group 3; +ve mode), n = 8 (group 3; -ve mode), n = 8 (group 5), n = 7 (group 6), n = 8 (group 8). PC, phosphatidylcholine; PE, phosphatidylethanolamine. Source data are provided as a Source Data file.

A

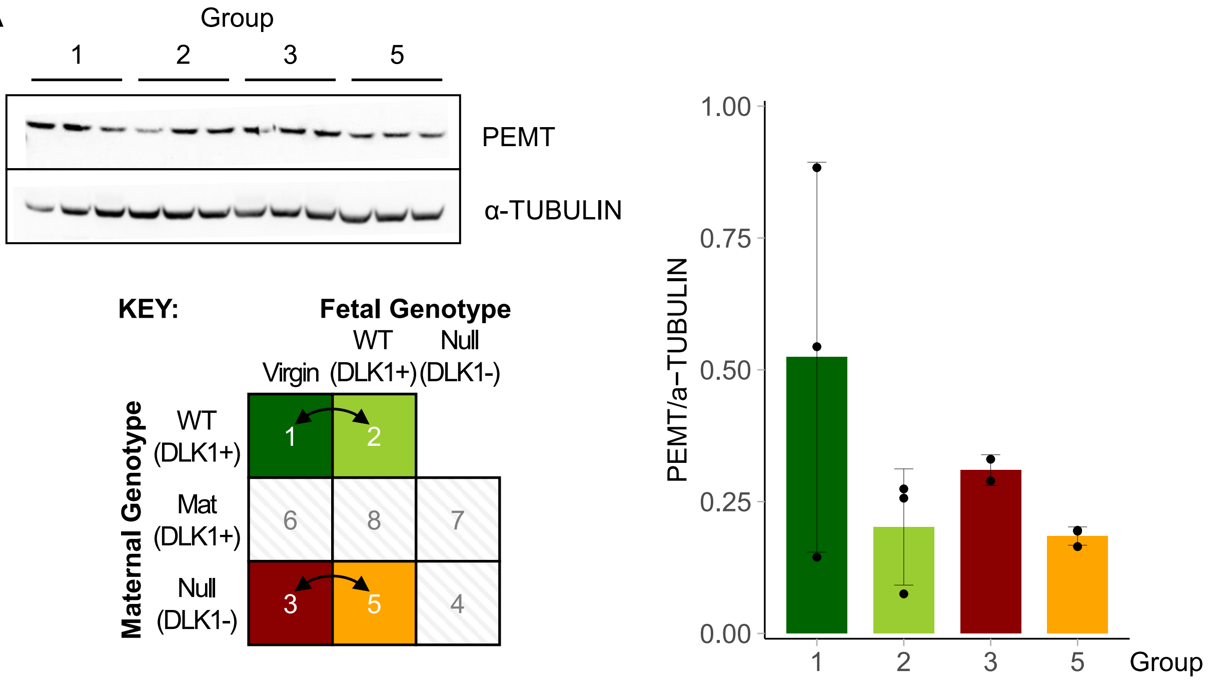
Group
1
2
3
5
PEMT
α-TUBULIN
KEY:
Fetal Genotype
Virgin
WT (DLK1+)
Null (DLK1-)
Maternal Genotype
WT (DLK1+)
Mat (DLK1+)
Null (DLK1-)
1 2 6 8 7 3 5 4
PEMT/a−TUBULIN
1.00
0.75
0.50
0.25
0.00
Group

B

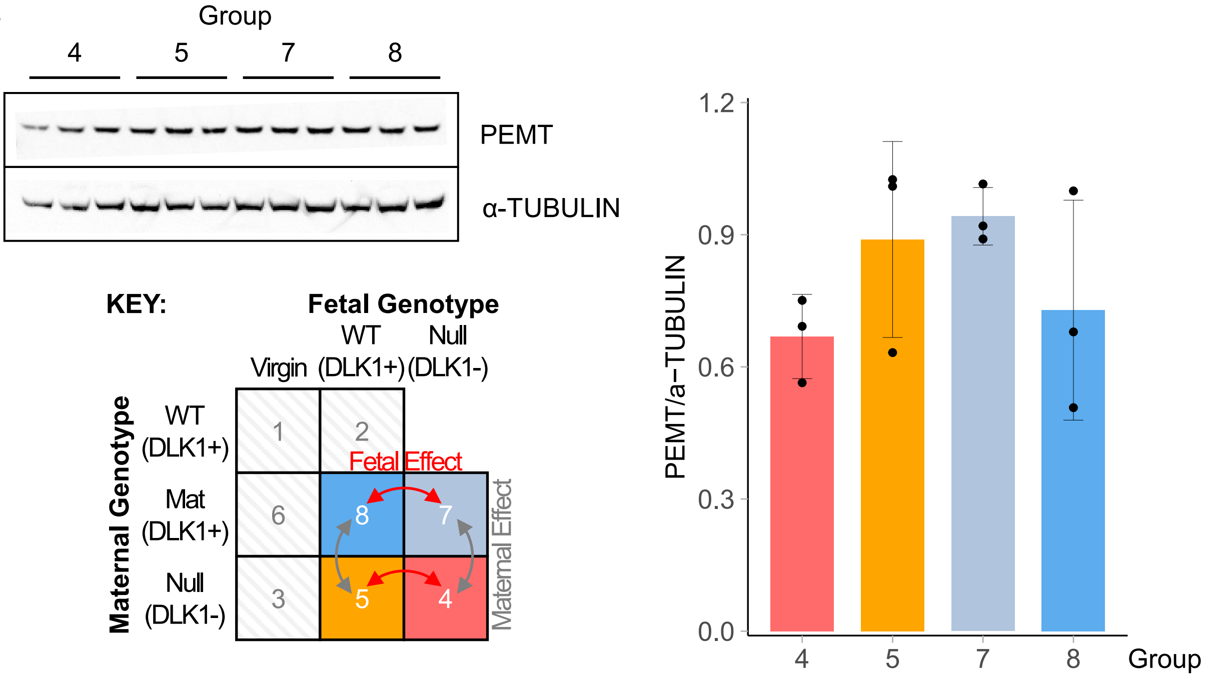
Group
4
5
7
8
PEMT
α-TUBULIN
KEY:
Fetal Genotype
Virgin
WT (DLK1+)
Null (DLK1-)
Maternal Genotype
WT (DLK1+)
Mat (DLK1+)
Null (DLK1-)
Fetal Effect
Maternal Effect
1 2 6 8 7 3 5 4
PEMT/a−TUBULIN
1.2
0.9
0.6
0.3
0.0
Group

C

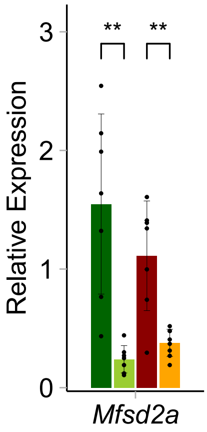
Relative Expression
3
2
1
0
**
**
Mfsd2a

Figure S4: (A-B) Western blotting of PEMT protein levels from liver lysates of two genotype-matched virgin vs pregnant (15.5 dpc) group comparisons **(A)** and in the four pregnant groups used to assess the effect of maternal-derived DLK1 protein or fetal-derived DLK1 protein in pregnancy **(B)**. PEMT bands are normalised to the housekeeping protein α-tubulin. n = 3 biological replicates/group. Uncropped blot images are supplied at the end of the Supplementary Information file. **(C)** Real-time quantitative PCR (RT-qPCR) of lyso-phospholipid transporter, *Mfsd2a,* in livers from two genotype-matched virgin vs pregnant group comparisons. Data was normalised to housekeeping gene expression (*Tuba1*, *Tbp* and *Hprt*) and is shown as mean relative expression ± SD. Groups were called significantly different by two-tailed Mann-Whitney U tests (* p-value <0.05; ** p-value <0.01; *** p-value <0.001). p-values for *Mfsd2a*: 1vs2 = 0.0012, 3vs5 = 0.0042. n = 8 (group 1), n = 7 (group 2), n = 8 (group 3), n = 8 (group 5); mice per group. Source data are provided as a Source Data file.

Supplementary Figure 5

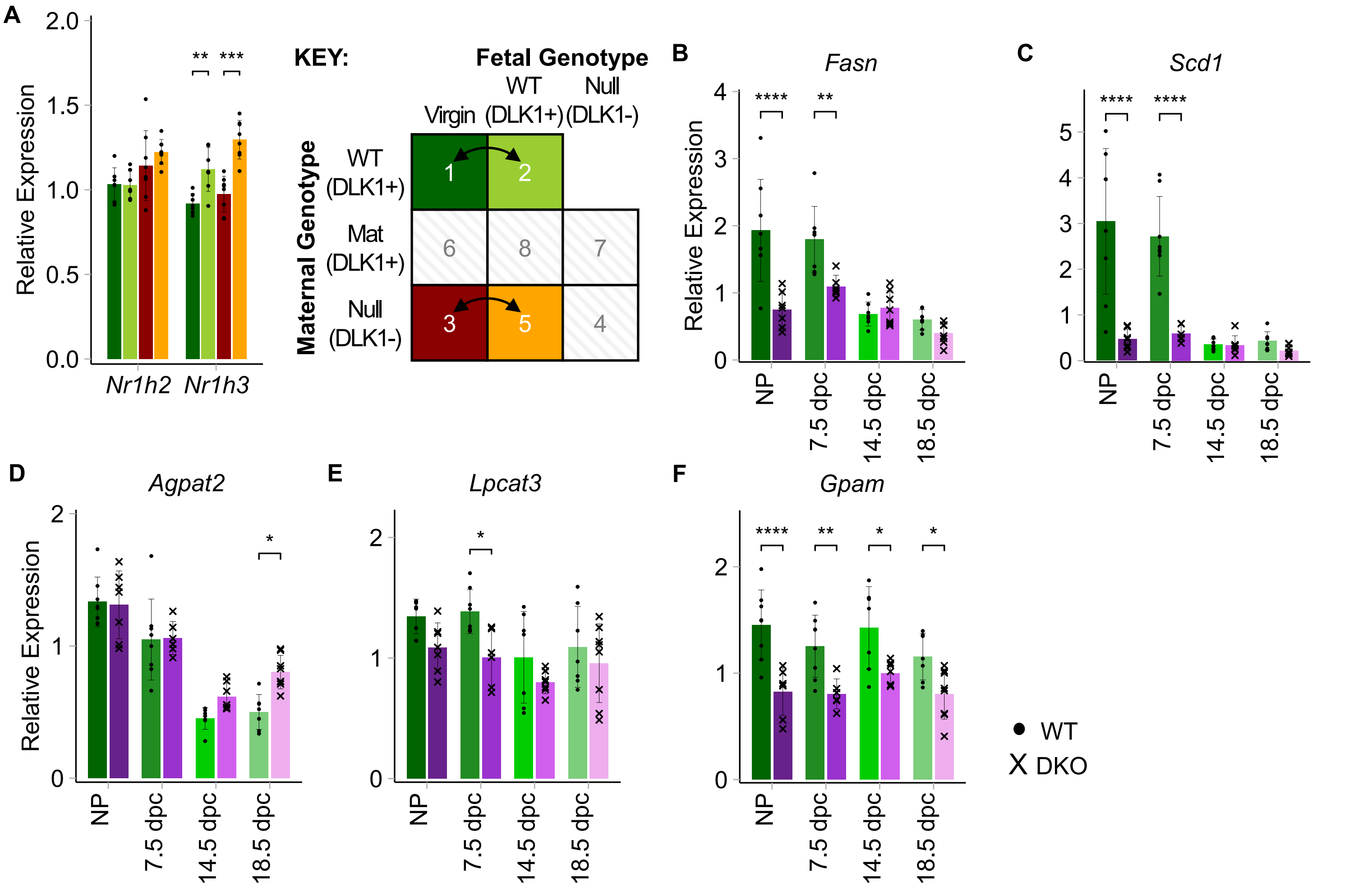

Figure S5: (A) Real-time quantitative PCR (RT-qPCR) analysis of liver X receptor (LXR) isoforms, LXRα (*Nr1h3*) and LXRβ (*Nr1h2*), in livers from two genotype-matched virgin vs pregnant (15.5 dpc) group comparisons. n = 8 (group 1), n = 7 (group 2), n = 8 (group 3), n = 8 (group 5); mice per group. **(B-F)** RT-qPCR analysis of classical lipogenic LXR target genes **(B-C)** and additional LC-PUFA-phospholipid biosynthetic genes **(D-F)** in an independent cohort of wild-type (WT) and *Lxrab*$^{-/-}$ (LXR double knockout (DKO)) mice at non-pregnant and various gestational timepoints. n = 8 (NP WT), n = 7 (NP DKO), n = 8 (7.5 dpc WT), n = 6 (7.5 dpc DKO), n = 7 (14.5 dpc WT), n = 7 (14.5 dpc DKO) n = 7 (18.5 dpc WT), n = 8 (18.5 dpc DKO); mice per condition. RT-qPCR data was normalised to housekeeping gene expression (*Tuba1*, *Tbp* and *Hprt*) and is shown as mean relative expression ± SD. Virgin vs pregnant groups were compared by two-tailed Mann-Whitney U tests, WT vs LXR DKO groups were compared by two-way ANOVA with Šídák's multiple comparison (* p-value <0.05; ** p-value <0.01; *** p-value <0.001; **** p-value <0.0001). p-values for *Nr1h3*: 1vs2 = 0.007, 3vs5 = 0.0003. WT vs DKO p-values for *Fasn*: NP = 8.47x10^{-07}, 7.5 dpc = 0.0049; *Scd1*: NP = 1.35x10^{-08}, 7.5 dpc = 2.96x10^{-06}; *Agpat2*: 18.5 dpc = 0.0106; *Lpcat3*: 7.5 dpc = 0.03; *Gpam*: NP = 9.87x10^{-05}, 7.5 dpc = 0.01, 14.5 dpc = 0.0132, 17.5 dpc = 0.0469. Source data are provided as a Source Data file.
