# Supplementary material for: A co-ordinated transcriptional programme in the maternal liver supplies long chain polyunsaturated fatty acids to the conceptus using phospholipids
Source: Nat Commun. 2024 Aug 8;15:6767. doi: 10.1038/s41467-024-51089-z (PMC11310303; doi:10.1038/s41467-024-51089-z)
Supplement: Supplementary file 3 — Description of Additional Supplementary Files [file 41467_2024_51089_MOESM3_ESM.docx]

# **Description of Additional Supplementary Files**

# **Supplementary Data Tables**

**Tables S1-2:** Mean relative abundance of all measured lipid classes for the 8 experimental groups in plasma (Table S1) and liver (Table S2). Two-way ANOVA with Sidak’s multiple comparisons tests were performed to determine significant class shifts between three genotype-matched virgin vs pregnant (15.5 dpc) comparisons, two genotype-matched pregnant groups with vs without a functional fetal-derived DLK1 protein (“Fetal Effect”) and two genotype-matched pregnant groups with vs without DLK1 sourced from maternal tissues (“Maternal Effect”; * p-value <0.05; ** p-value <0.01; *** p-value <0.001; **** p-value <0.0001). Statistical tests were performed independently per ionisation mode and per genotype-matched replicate comparison. Plasma data: n = 6 (group 1), n = 7 (group 2), n = 7 (group 3), n = 5 (group 4; +ve mode) n = 8 (group 4; -ve mode), n = 6 (group 5), n = 5 (group 6), n = 8 (group 7), n = 6 (group 8; +ve mode), n = 7 (group 8; -ve mode); Liver data: n = 7 (group 1), n = 6 (group 2), n = 7 (group 3), n = 8 (group 4), n = 8 (group 5), n = 7 (group 6), n = 7 (group 7; +ve mode), n = 8 (group 7; -ve mode), n = 8 (group 8); mice per group. Source data are provided as a Source Data file.

**Table S3:** Phosphorus nuclear magnetic resonance (31P NMR) class profile of pooled livers samples from virgin (groups 1, 3 and 6) and pregnant mice at 15.5 dpc (groups 2, 5 and 8). CL, cardiolipin; LPC, Lyso- phosphatidylcholine; PC, phosphatidylcholine; PE, phosphatidylethanolamine; PI, phosphatidylinositol; SM, sphingomyelin. Source data are provided as a Source Data file.

**Tables S4-5:** Lipids identified as candidate biomarkers (CBMs) that best distinguished pregnant from virgin groups (Table S4) and pregnant groups lacking maternal-derived DLK1 from those with normal expression of maternal-derived DLK1 (Table S5). CBMs were classified as lipids that passed both Bonferroni-adjusted two-tailed *t*-tests (liver threshold, p = 0.00234; plasma threshold, p = 0.00283) and sparse partial least squares discriminant analysis in at least two genotype-matched replicate comparisons. Data is presented as mean relative abundance (±SD) per ionisation mode. All CBM tests were performed independently per ionisation mode and per genotype-matched replicate comparison. Plasma data: n = 7 (group 1), n = 7 (group 2), n = 7 (group 3), n = 5 (group 4; +ve mode), n = 8 (group 4; -ve mode), n = 6 (group 5), n = 5 (group 6), n = 8 (group 7), n = 7 (group 8); Liver data: n = 8 (group 1; +ve mode), n = 7 (group 1; -ve mode), n = 7 (group 2), n = 7 (group 3; +ve mode), n = 8 (group 3; -ve mode), n = 8 (group 4), n = 8 (group 5), n = 7 (group 6), n = 8 (group 7), n = 8 (group 8); mice per group. Source data are provided as a Source Data file.

**Table S6:** LC-MS/MS analysis of liver and plasma for the presence and fatty acid composition of select PC and PE lipids. Data is presented as % total lipid/glyceride signal. The MS2 spectrum in the positive mode was used to identify PC lipids and the MS2 spectrum in the negative mode was used to identify PE lipids. Undetermined values were below the limit of detection’. PC, phosphatidylcholine; PE, phosphatidylethanolamine. Source data are provided in a public repository.

**Table S7:** List of lipids included in Figures 3E, F. The most commonly-occurring ARA and DHA-containing PC and PE lipids were selected using a published lipidomics profile of the mouse liver (Taguchi et al. 2010). *sn*-1/*sn*-2 fatty acid compositions are annotated using the most abundant isoform identified from targeted LC-MS/MS analyses in plasma and liver (percentage abundance indicated in brackets; see Supplementary Table S6). CBMs were classified as lipids that passed both Bonferroni-adjusted two-tailed *t*-tests (liver threshold, p = 0.00234; plasma threshold, p = 0.00283) and sparse partial least squares discriminant analysis in at least two genotype-matched virgins vs pregnant group comparisons. All CBM tests were performed independently per ionisation mode and per genotype-matched replicate comparison. PC lipids were selected from the positive mode while PE lipids were selected from the negative mode. ARA, arachidonic acid; DHA, docosahexaenoic acid; PC, phosphatidylcholine; PE, phosphatidylethanolamine. Source data are provided as a Source Data file.

**Table S8:** Mean concentrations of all metabolites measured in the PUFA metabolite panel. Metabolites were measured in livers from all 8 experimental groups. Metabolite names are defined in the Materials and Methods. n = 8 (group 1), n = 7 (group 2), n = 8 (group 3), n = 8 (group 4), n = 8 (group 5), n = 7 (group 6), n = 8 (group 7), n = 8 (group 8); mice per group. Source data are provided as a Source Data file.

**Table S9:** Differentially expressed genes (DEGs) generated from the re-analysis of a published microarray dataset that compared liver transcriptomes between virgin and pregnant mice at 14.5 dpc (n = 4 mice per condition; Quinn et al. 2019). Differential expression analysis was performed using limma with a p-value (corrected for multiple hypothesis testing based on the Benjamini–Hochberg procedure) threshold of 0.05 and a fold change threshold of 1.25.

**Table S10:** Genes in candidate pathways of interest selected from the re-analysed microarray dataset that compared liver transcriptomes between virgin and pregnant mice at 14.5 dpc (n = 4 mice per condition; Quinn et al. 2019). Differential expression analysis was performed using limma with a p-value (corrected for multiple hypothesis testing based on the Benjamini–Hochberg procedure) threshold of 0.05 and a fold change threshold of 1.25.

**Table S11:** Significant transcription factor pathways resulting from the overlap analysis between transcription factor targets and upregulated differentially expressed genes (DEGs) generated from the re-analysis of a published whole-transcriptome microarray dataset of livers from pregnant and virgin mice (n = 4 mice per condition; Quinn et al. 2019), using the ChIP enrichment analysis (ChEA) through the Enrichr platform. All overlapping gene names are indicated for each pathway. Differential expression analysis was performed using limma with a p-value (corrected for multiple hypothesis testing based on the Benjamini–Hochberg procedure) threshold of 0.05 and a fold change threshold of 1.25 (DEG list is found in Supplementary Table S9).

**Table S12:** Mean concentrations of select LC-PUFA-containing lipid species measured by targeted LC-MS/MS in an independent cohort of wild-type (WT) and *Lxrab*^−/−^ (LXR double knockout (DKO)) mice at late-gestational timepoints. Liver and serum were measured in 14.5 dpc and 18.5 dpc dams, and placenta and fetal livers were analysed from 18.5 dpc dams only. Bonferroni-adjusted two-tailed *t*-tests (p-value threshold = 0.025) were performed for each WT vs DKO comparison. n = 7 (14.5 dpc WT), n = 7 (14.5 dpc DKO), n = 7 (18.5 dpc WT; exceptions: n=6 for placenta), n = 8 (18.5 dpc DKO; exceptions: n=6 for placenta; n = 7 for fetal liver); mice per condition. ARA, arachidonic acid; DHA, docosahexaenoic acid; PC, phosphatidylcholine; PE, phosphatidylethanolamine. Source data are provided as a Source Data file.

**Table S13:** Macronutrient and fatty acid composition of the RM3 standard chow diet fed to the animals in the current study.

**Table S14:** List of internal standards used for lipid profiling.

**Table S15:** List of lipid species and known *m/z* values that were measured in the positive and negative ionisation modes by direct-infusion mass spectrometry.

**Table S16:** RT-qPCR primer sequences and sources.
