# Supplementary material for: A co-ordinated transcriptional programme in the maternal liver supplies long chain polyunsaturated fatty acids to the conceptus using phospholipids
Source: Nat Commun. 2024 Aug 8;15:6767. doi: 10.1038/s41467-024-51089-z (PMC11310303; doi:10.1038/s41467-024-51089-z)
Supplement: Supplementary file 5 — Reporting Summary [file 41467_2024_51089_MOESM5_ESM.pdf]

nature portfolio

Corresponding author(s): M Charalambous

Last updated by author(s): 2024/04/16

Reporting Summary

Nature Portfolio wishes to improve the reproducibility of the work that we publish. This form provides structure for consistency and transparency in reporting. For further information on Nature Portfolio policies, see our Editorial Policies and the Editorial Policy Checklist.

Statistics

For all statistical analyses, confirm that the following items are present in the figure legend, table legend, main text, or Methods section.

n/a	Confirmed	
☐	☒	The exact sample size (*n*) for each experimental group/condition, given as a discrete number and unit of measurement
☐	☒	A statement on whether measurements were taken from distinct samples or whether the same sample was measured repeatedly
☐	☒	The statistical test(s) used AND whether they are one- or two-sided<br>*Only common tests should be described solely by name; describe more complex techniques in the Methods section.*
☒	☐	A description of all covariates tested
☐	☒	A description of any assumptions or corrections, such as tests of normality and adjustment for multiple comparisons
☐	☒	A full description of the statistical parameters including central tendency (e.g. means) or other basic estimates (e.g. regression coefficient) AND variation (e.g. standard deviation) or associated estimates of uncertainty (e.g. confidence intervals)
☐	☒	For null hypothesis testing, the test statistic (e.g. *F*, *t*, *r*) with confidence intervals, effect sizes, degrees of freedom and *P* value noted<br>*Give P values as exact values whenever suitable.*
☒	☐	For Bayesian analysis, information on the choice of priors and Markov chain Monte Carlo settings
☒	☐	For hierarchical and complex designs, identification of the appropriate level for tests and full reporting of outcomes
☐	☒	Estimates of effect sizes (e.g. Cohen's *d*, Pearson's *r*), indicating how they were calculated

Our web collection on statistics for biologists contains articles on many of the points above.

Software and code

Policy information about availability of computer code

Data collection	No software was used for data collection.
Data analysis	XCMS (www.bioconductor.org), Peakpicker 2.0(an in-house R script), MassHunter Quantitative analysis software (Agilent Technologies), Transcriptome Analysis Console software (4.0.2; ThermoFisher), EnrichR (maayanlab.cloud/Enrichr/), Primer3 (primer3.ut.ee), Excel (Office 365), MetaboAnalyst 4.0, GraphPad Prism Software (8.4.3) for Windows (www.graphpad.com). R (version 4.1.1), EnhancedVolcano/Bioconductor package (version 3.15).

Data

Policy information about availability of data

All lipidomics datasets generated in this study have been deposited in the EMBL-EBI MetaboLights database DOI: 10.1093/nar/gkad1045, PMID:37971328) with the identifier MTBLS9901. The NMR data used in this study are available in the EMBL-EBI BioStudies database under accession code S-BSST651 [https://www.ebi.ac.uk/biostudies/bioimages/studies/S-BSST651]. The processed PUFA metabolite data generated in this study is provided as a source data file. The microarray dataset re-analysed in this study is available on the Gene Expression Omnibus under accession code GSE121202 [https://www.ncbi.nlm.nih.gov/geo/query/acc.cgi?acc=GSE121202]. Source data are provided with this paper.

Research involving human participants, their data, or biological material

Policy information about studies with human participants or human data. See also policy information about sex, gender (identity/presentation), and sexual orientation and race, ethnicity and racism.

Reporting on sex and gender	n/a
Reporting on race, ethnicity, or other socially relevant groupings	n/a
Population characteristics	n/a
Recruitment	n/a
Ethics oversight	n/a

Field-specific reporting

Please select the one below that is the best fit for your research. If you are not sure, read the appropriate sections before making your selection.

☒ Life sciences ☐ Behavioural & social sciences ☐ Ecological, evolutionary & environmental sciences

For a reference copy of the document with all sections, see nature.com/documents/nr-reporting-summary-flat.pdf

Life sciences study design

All studies must disclose on these points even when the disclosure is negative.

Sample size	Sample sizes were determined based on power calculations performed on data from previous studies performed by the authors.
Data exclusions	No data was excluded except for in the Lipidomics analysis where Principal component analyses (PCA) were first performed to identify and exclude sample outliers based on 95% confidence intervals.
Replication	For all comparisons between experimental cohorts, significance was only considered if identified in at least two genotype-matched replicate comparisons. Findings from the comparisons of non-pregnant and pregnant mice from the main experimental cohort were replicated in two independent mouse cohorts. All replication was successful.
Randomization	Animals were randomly assigned to different experimental groups.
Blinding	Blinding was not performed in this study. However, all data and tisssue collection was performed without reference to genotype. Pregnant vs non-pregnant groups cannot be blinded since at data collection the group is macroscopically obvious.

Reporting for specific materials, systems and methods

We require information from authors about some types of materials, experimental systems and methods used in many studies. Here, indicate whether each material, system or method listed is relevant to your study. If you are not sure if a list item applies to your research, read the appropriate section before selecting a response.

Materials & experimental systems

n/a	Involved in the study	
☐	☒	Antibodies
☒	☐	Eukaryotic cell lines
☒	☐	Palaeontology and archaeology
☐	☒	Animals and other organisms
☒	☐	Clinical data
☒	☐	Dual use research of concern
☒	☐	Plants

Methods

n/a	Involved in the study	
☒	☐	ChIP-seq
☒	☐	Flow cytometry
☒	☐	MRI-based neuroimaging

Antibodies

Antibodies used

Rabbit anti-human COX1 (1:200, ab133319 abcam, lot#GR98049_11); Rat anti-mouse Endomucin, (1:200, sc-65495 Santa Cruz, lot#D1117); Texas red-conjugated goat anti-rat (1:300, TI-9400 Vector Labs, lot#W0322); Goat anti-rabbit DyLight® 488 (1:300, DI1488 Vector Laboratories, lot#Z0514); Rabbit anti-PEMT polyclonal antibody (1:1000, Bio-techne #NBP1-59580, lot#QC12965-150403); Mouse anti-α-TUBULIN monoclonal antibody (1:10,000, Millipore-SIGMA #T5168, lot#035M4878V); Goat anti-rabbit (1：1000, P0448 DAKO, lot#20066477); Goat anti-mouse (1：2000, P0447 DAKO, lot#20062696).

Validation

https://www.abcam.com/products/primary-antibodies/cox1--cyclooxygenase-1-antibody-epr5867-ab133319.html; https://www.scbt.com/p/endomucin-antibody-v-7c7; https://www.novusbio.com/products/pemt-antibody_nbp1-59580; https://www.sigmaaldrich.com/GB/en/product/sigma/t5168

Animals and other research organisms

Policy information about studies involving animals; ARRIVE guidelines recommended for reporting animal research, and Sex and Gender in Research

Laboratory animals

[illegible]

Wild animals

none

Reporting on sex

The majority of work was performed on pregnant mice using virgin female littermates as controls. Since the study is primarily concerned with maternal metabolism this is appropriate.

Field-collected samples

No field collected samples were used in this study.

Ethics oversight

All animal experiments were carried out with approval of the UK Government Home Office licencing procedures under licence P24AB281B awarded to MC and were approved by local animal ethics committees at the University of Cambridge or King's College London.

Plants

Seed stocks

n/a

Novel plant genotypes

n/a

Authentication

n/a
